# Augmented-Reality Presentation of Household Sounds for Deaf and Hard-of-Hearing People

**DOI:** 10.3390/s23177616

**Published:** 2023-09-02

**Authors:** Takumi Asakura

**Affiliations:** Department of Mechanical and Aerospace Engineering, Faculty of Science and Technology, Tokyo University of Science, Chiba 278-8510, Japan; t_asakura@rs.tus.ac.jp

**Keywords:** deaf and hard-of-hearing (DHH) people, augmented reality, environmental sound recognition, machine learning, dynamic spectrogram

## Abstract

Normal-hearing people use sound as a cue to recognize various events that occur in their surrounding environment; however, this is not possible for deaf and hearing of hard (DHH) people, and in such a context they may not be able to freely detect their surrounding environment. Therefore, there is an opportunity to create a convenient device that can detect sounds occurring in daily life and present them visually instead of auditorily. Additionally, it is of great importance to appropriately evaluate how such a supporting device would change the lives of DHH people. The current study proposes an augmented-reality-based system for presenting household sounds to DHH people as visual information. We examined the effect of displaying both the icons indicating sounds classified by machine learning and a dynamic spectrogram indicating the real-time time–frequency characteristics of the environmental sounds. First, the issues that DHH people perceive as problems in their daily lives were investigated through a survey, suggesting that DHH people need to visualize their surrounding sound environment. Then, after the accuracy of the machine-learning-based classifier installed in the proposed system was validated, the subjective impression of how the proposed system increased the comfort of daily life was obtained through a field experiment in a real residence. The results confirmed that the comfort of daily life in household spaces can be improved by combining not only the classification results of machine learning but also the real-time display of spectrograms.

## 1. Introduction

### Background and Previous Research

According to the World Health Organization, there are more than 360 million deaf and hard-of-hearing (DHH) people worldwide who are considered to have partial or complete hearing impairment [1]. These DHH people have difficulty hearing sounds in their surroundings. In particular, visual and auditory information is used for various environmental perceptions, the importance of which varies from situation to situation [2].

In such cases where either visual or auditory information is missing, it may not be possible to obtain sufficient environmental information needed for the comfort, safety, and security of human life. In such a situation, DHH people have difficulty receiving environmental sound information, which makes it difficult for them to perform environmental sound recognition (ESR) in their everyday life and make decisions based on auditory information, as is the case for people with normal hearing. For some cochlear implant patients, environmental rehabilitation may be beneficial [3], with the potential for low-cost and effective rehabilitation using ESR [4], and the application of sound visualization techniques using onomatopoeia [5] have been developed. Pick-by-Vision, which uses smart glasses to visually assist order pickers in their work tasks, has also been demonstrated [3,4,5,6].

Various environmental sound classification (ESC) technologies based on machine learning (ML) have been investigated in recent years as an alternative to human perception of environmental sounds. One review [7] suggested that the benefits of ESC are the development of hearing aids, crime investigation, and security systems, and that ML-based ESC can be largely applied to industry. ML classifiers include support vector machines [8], K-nearest neighbor [8], decision trees [9], and hidden Markov models [10]. In addition, novel methods such as convolutional neural networks [11], multilayer perceptrons [12], and recurrent neural networks [13] are used extensively.

To enhance DHH people’s hearing ability, hardware that incorporates algorithms for the various ESR techniques described above is being developed. For example, some technologies can facilitate communication between sign-language users and non-sign-language users, such as the technology to convert sign language into linguistic information using Android terminals [14] and mobile terminals that recognize gestures by using smart gloves [15]. There are also technologies to support communication between differently abled people and those with visual, auditory, or speech impairments by converting speech signals into textual data [16]. Furthermore, we can also find studies (e.g., [17]) that consider wearable devices that notify DHH people by converting environmental sounds into vibration. There are also examples of the development of systems that notify users of such environmental sounds by means of smartphones [18]. In addition, technology has been developed [19] in which subtitles representing the speaker’s words are provided in real time on augmented-reality (AR) displays [19]. Ribeiro et al. [20] produced natural maps of visual and auditory modalities by using technology that maps environmental sounds as virtual objects on a head-mounted display, allowing for intuitive representation. In addition, a case study has been conducted to investigate user preferences regarding wearable or smart device environmental sound presentation technologies [21]. The visualization of auditory information has been investigated in various fields, including the study of physically visualizing the intensity of sound generated by sound sources using a see-through head-mounted display [22], as well as in music, education, consumer electronics, marine science, medicine, and ecosystems [23,24,25,26,27,28]. By visualizing sound, it may be possible to obtain a variety of information that cannot be obtained simply by hearing. A number of technologies have been proposed to support the understanding of the sound environment by visualizing sounds for DHH people. Various studies have been carried out to visualize, for example, music. Basic music visualizations include 2D time-series displays (e.g., waveforms and spectrograms) for characteristics such as pitch or loudness [29,30,31,32]. For DHH users specifically, Nanayakkara et al. [33] created an abstract display that changed size, color, and brightness in step with the harmonics to enhance the musical experiences of DHH people. New York City designed a wall display to convey musical frequencies through light to teach music to DHH children [34].

Much research has been conducted on the visualization of environmental sounds. In particular, some studies have investigated the visualization of common sounds by using various smart devices to support DHH people [35,36,37,38,39,40,41,42,43]. As quite a practical study, Jain et al. [42] investigated HomeSound, which visualizes sound to provide awareness of household sounds inside the homes of DHH people, suggesting a variety of preferable conditions to display form factors such as smartphones or tablets, sound types such as alarms and alerts, and sound characteristics such as location and time duration. Matthews et al. [35] explored the requirements for the functionality of environmental sound visualization based on feedback from DHH people and used several visualization systems, including spectrograms, to enable an understanding of environmental sounds that are useful to people with hearing impairment.

In light of these previous studies, various technologies for visualizing sound have been proposed to support people with hearing disabilities. Among these, the spectrogram, which can visualize both the time and frequency characteristics of sound, has benefits, but there have not been many examples of the visualization of a wide range of environmental sounds using spectrograms for the purpose of supporting DHH people.

Spectrograms have been used by DHH people to recognize speech [44,45,46,47,48,49,50,51]. The first attempts to read speech from spectrograms were made by Potter et al. [44], with limited success. In recent years, Hillier et al. [51] proposed a modified spectrogram that incorporated increased frequency resolution and the enhancement of important sound information, resulting in improved word recognition from 23% to 80%. The spectrogram has been gradually increasingly used in hearing-aid technology, but visualization technologies based on the spectrogram remain in the developmental stage and cannot yet adequately support DHH people.

Normal-hearing people use sound as a cue to recognize various sound-related events that occur in their surrounding environment. However, it is difficult for DHH people to freely detect the surrounding sound environment. Therefore, the purpose of the present study is first to create a convenient device that can detect sounds occurring in daily life and present the detected sound-related events as visually related measures. Secondly, how the created supporting device could change the lives of DHH people is appropriately evaluated from the viewpoint of increasing QOL.

To achieve the above two objectives, this study first extracts the sounds of daily life that DHH people have trouble hearing in their dwellings, based on the results of interviews and previous research, and then constructs an AR-based presentation system that can convert these sounds from the dwellings into visual information in real time. This system stands on the following two techniques: one is an ESR technique using machine learning and the other is an active method of ESR by the users themselves using spectrograms, which has been identified as useful in previous studies but has not yet reached the practical stage of knowledge. The system enables the detection of what kinds of sounds are being generated, and whether the successful detection reduces the stress of the user and ultimately provides comfortability of daily life, which were examined in an onsite subjective evaluation experiment.

## 2. Research Flow and Basic Survey on Background of Research Problem

### 2.1. Purpose and Research Flow

**Figure 1:**
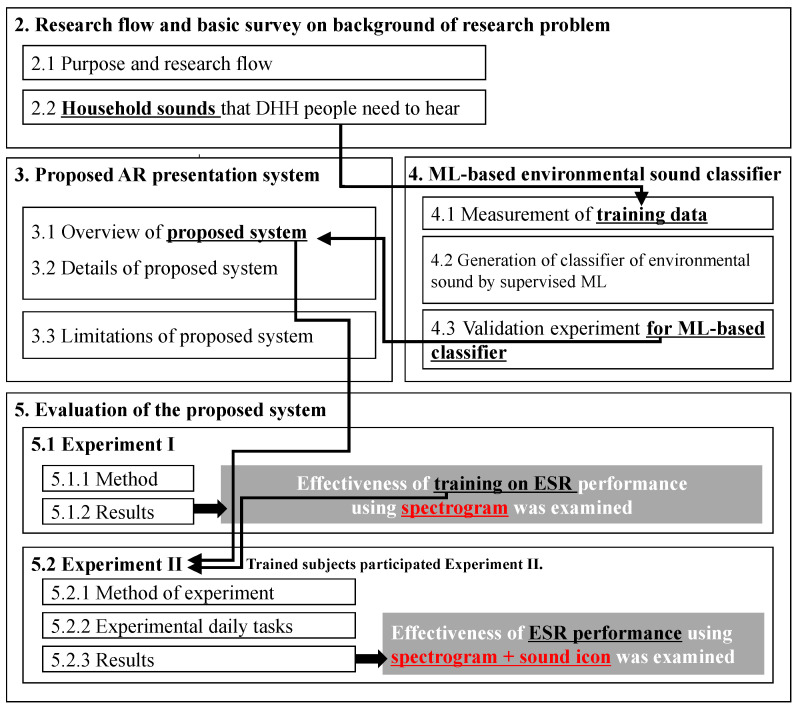
Flowchart of entire sections.

The present paper is organized into the following five sections. The relationship between the investigated content in each of the sections is indicated in Figure 1. First, Section 2 describes how the issues that DHH people perceive as problems in their daily lives were investigated through a hearing survey of current and previous studies. Then, Section 3 describes the proposed system that is designed to display a dynamic spectrogram in real time as well as icons indicating the surrounding environmental sounds judged by an ML classifier. In this proposed system, the trained classifier for the target household sounds that are suggested as the key sounds in Section 2 are installed. So, the ML-based classifier for the abovementioned target household sounds is generated in Section 4 and installed in the proposed system. Then, the effectiveness of the proposed method is verified in Section 5. It should be noted that the natural use of the dynamic spectrogram to analogize “what the sound is” requires training. So, before the main Experiment II, Experiment I in Section 5.1 examines whether training to accurately estimate the sound from the dynamic spectrogram is effective, showing that three replicates of training and confirmation trials improved the accuracy of sound recognition. Then, all the trained subjects participated in Experiment II in Section 5.2 where the ESR performance using the presentation system with and without the spectrogram was comparatively discussed through subjective impressions, including ease of use and perceived comfortability.

### 2.2. Survey on Daily Problems of DHH People

To understand the actual situation regarding the relationship between DHH people and environmental sounds in their daily lives, DHH people were surveyed through interviews. The questions of the survey as addressed the following three issues:

1. What are the environmental sounds that are difficult to hear in their daily lives?
2. What are the problems of not being able to hear the abovementioned sounds?
3. How do people obtain or not obtain information on surrounding sounds?

The current interviews were conducted as a focus group interview [52] against three subjects with post-lingual hearing loss via sign-language interpreters. As the number of subjects in the current interview provided qualitative interview [53] results, the quantitative aspect of this survey was supplemented by a multifaceted comparison with previous studies. Specifically, reference [54], in which the abovementioned problems in daily life were investigated through a large survey conducted with deaf people. The survey in this reference investigated the difficulties caused by hearing impairment in various required activities between waking up in the morning and going to bed at night. The main issues that were most frequently mentioned by respondents were extracted. It should be noted that in the above reference (ICCD 1995) [54], questionnaire response data were obtained for the respondents belonging to a total of four categories regarding the degree of hearing impairment: A: able to hear sounds with the bare ear; B: able to somewhat hear sounds with the bare ear; C: unable to hear sounds at all; D: degree of hearing ability unknown. Out of a total of 228 data points, the results of the 180 respondents who chose C were cited. It should be noted that the proportion of male and female respondents in this reference was 34.7% and 65.3%, respectively, and the ages of the respondents were distributed over a wide range, with the most common age group being the 50s, representing 24.5% of all the respondents. The proportion of hearing-aid users was 61.4%. In addition to this reference, other papers [18,36,43,55,56] were also cited to enhance the results.

Table 1 summarizes the comments raised in response to the above three questions asked in the interview of the current investigation and the previous references. In this table, each issue is classified into the following categories: general issues, interior issues, exterior issues, and interior and exterior issues. Additionally, the relevant sub-categories are mainly classified into “auditory detection”, “visual detection” and other sub-categories such as “detecting events”, “communication” and “reading text”. Then, the check marks are assigned to each of the references where the corresponding comments are described. In addition, the column of reference [54] includes the comment contents with the number of respondents in addition to the check marks, while the rightmost column shows the other references that had similar comments.

First, three comments on the issues from a general perspective of daily life are shown. One is that DHH people have trouble detecting sound-related events, while relatedly, they also have difficulty communicating with others. The former issue was also mentioned by Jain et al. [42]. Moreover, as a comment related to communication with others, it was noted that reading text is difficult for people with a natural hearing impairment. In reference [57], similar difficulties reading texts are also mentioned for DHH people. Therefore, many DHH people use sign language [58] and lip-reading [59] as tools for interpersonal communication. As these are the main languages of people born with hearing impairment, the language and grammar they acquire are unique to sign language, and they are likely to have difficulties with reading and writing [60], as well as understanding the sentences of people with normal hearing when writing. Therefore, it is also said that they may also have difficulty converting speech into language and understanding it.

Next, 11 issues related to the interior events were commented on. Among these, a comment related to visual issues was made, as well as auditory issues. There are sound-related events that are difficult to detect by DHH people. For example, it is difficult for them to wake up on time because they cannot hear alarm sounds, which was commented by 45 people among the 180 in the reference [54]. These are also suggested by past research. Difficulties cooking were also mentioned in previous studies, but not in the current interview. The previous study also indicated that they miss sound cues from appliances such as kettles, microwave ovens, and smoke detectors. They also made comments such as “I have left the vacuum cleaner running all night” [35]. The previous study in [54] also indicated comments on the difficulties related to running water (23/180), washing machines (41/180), and vacuum cleaners (11/180). Related to these issues, the current interview also indicated the frustration of not being able to notice familiar environmental sounds, such as the water-running sound because of forgetting to turn off the tap. Additionally, the current interview showed that the subject failed to take in the laundry that was hanging outdoors when it started to rain. On the other hand, some issues related to people other than themselves were suggested by the current and previous interviews. These interviews showed an issue where DHH people cannot detect the sound of knocking on doors, while previous ones showed the difficulties detecting the doorbell sounds of someone coming home, the replying voice of someone, and a baby crying. Specifically, 54 respondents among the 180 in the previous interview (ICCD 1995) [54] suggested having difficulty detecting someone coming home. Related to this, Matthews et al. [35] stated that it is very inconvenient to wait for visitors because they cannot hear the knock on the door and have to visually check every few minutes, which is an important aspect in terms of communication with others. Unlike the auditory issues discussed above, the visual issue of using devices such as flashing lights as alternative methods to detect sound-related events is commented on in the current interview, while a similar comment about DHH people waking up using a vibration alarm instead of sounding alarm was made in all the studies. However, comments were also made in the current interview that such visual information is inconvenient because it cannot be noticed unless the object is within the user’s field of view.

Next, the exterior issues were described. It was commented in the current interview that they are unable to predict accidental events from information such as approaching vehicle sounds. In the previous literature, it has been reported that they feel unable to hear sounds related to hazard avoidance, such as emergency alarms, car horns, and the sound of approaching vehicles [55]. In addition, acquiring information on the exterior world through auditory information, such as noticing the sound of a vehicle approaching from behind, is important because it is one of the essential functions of hearing [61] and is an important aspect for DHH people to perceive the environment. In the previous interview [54], many problems in the exterior environment were commented on. For example, in addition to the above issue regarding the difficulty detecting approaching vehicles, other primary difficulties are observed in detecting public announcements (82/180) or a clerk’s call at the hospital (103/180) or banks or post offices (72/180), and communicating with a doctor in a hospital (79/180) or clerks in stores (70/180).

As the issues related to both auditory and visual information, the inability to detect the sound of someone’s approaching footsteps was mentioned in the current and previous interviews. In this case, people with unimpaired hearing can recognize that someone is approaching them and can take the next action, for example, to turn around and look. However, DHH people cannot hear footsteps, so they cannot realize that someone is approaching them and cannot react to the approach. Other difficulties using telephones or obtaining information in cases of disaster were also mentioned in the previous interview [54], though the number of the respondents was not large.

As mentioned above, in the interviews with DHH people, there was a great need to know about environmental sounds in the home as well as in workplaces or public places. This is thought to be due to the importance of being aware of the sound environment in the home, where the majority of daily life is spent, and living comfortably.

## 3. Proposed AR Presentation System

### 3.1. Overview of Proposed System

This study developed and validated a visual presentation system for environmental sounds. It displays both the results of environmental sound identification by ML as icons and a dynamic spectrogram from environmental sound analysis in real time. The icons and spectrograms are presented on smart glasses, and viewing this display allows the user to visually identify current environmental sounds. In this study, the effectiveness of the proposed system regarding the degree of recognition of environmental sounds occurring in a house was evaluated by conducting experiments simulating daily life with normal-hearing subjects who wore soundproof earmuffs to simulate hearing impairment.

Currently, it is not easy to accurately recognize environmental sounds using ML, which may lead to an accumulation of frustration for the user if classification results are based on incorrect recognition results. However, because the time and frequency characteristics of environmental sounds can be visualized with a spectrogram, a number of studies have attempted to detect spoken voice content from a spectrogram display. However, because environmental sounds are relatively difficult to recognize due to the problem of background noise [62] and the complexity of sound due to its rhythm and frequency characteristics, there have been few studies on techniques for estimating them from the spectrogram. Therefore, by presenting both icon and spectrogram displays of environmental sounds based on ML results, there is a possibility that the accuracy of the visual recognition of environmental sounds can be improved by having the two functions complement each other. Although spectrograms are difficult for ordinary users to understand, training can possibly also have an effect on ESR, as the recognition accuracy has been improved by training in the field of speech recognition [35,51,52,53,54,55,56,57]. Therefore, training on ESR was carried out in this study and its effects are discussed. Furthermore, an ESR experiment using the visualization system proposed in this paper was conducted on subjects whose recognition rate was improved by the training.

### 3.2. Details of Proposed System

**Figure 2:**
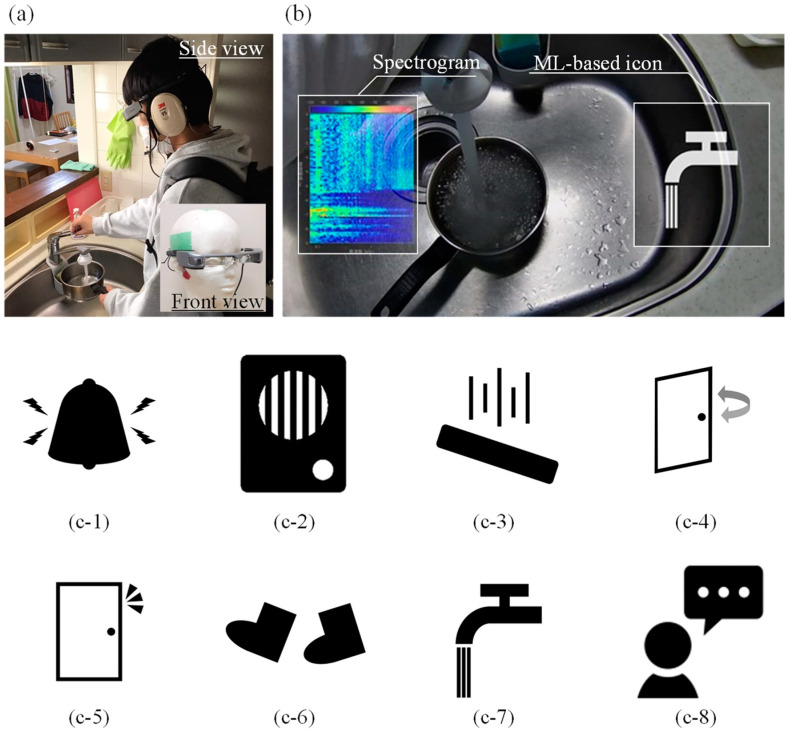
Overview of the proposed AR-based visual presentation system. (**a**) The subject cooking with the proposed system, (**b**) the AR view displayed on the smart glasses, and each of the icons for various sounds: (**c**-**1**) alarm, (**c**-**2**) intercom, (**c**-**3**) object dropping, (**c**-**4**) door opening or closing, (**c**-**5**) knocking on the door, (**c**-**6**) footsteps, (**c**-**7**) running water, and (**c**-**8**) human voice.

The appearance of the proposed system, the presented AR images including the ML-based icons and spectrograms on the display, and an example of the icons finally adopted in the validation experiment in Section 5 are shown in Figure 2a–c, respectively. As shown in Figure 2b, the subjects wear the smart glasses (Epson, BT-35E, Suwa, Japan) and perform the daily tasks described below while viewing the spectrogram and icons that change dynamically at every moment. To make it as easy as possible to obtain visual information in the field of view, the spectrogram is placed as close as possible to the left side and the icons to the right side. It should be noted that smart glasses have been used in various other fields of research [63,64,65,66] and are considered to be reliable in terms of their performance. For example, they have been used to assist in procedures such as bronchoscopy [63,64], needle biopsy for breast tumors [65], and in research cases aimed at very practical purposes such as completely different warehouse operations. The performance is expected to meet the requirements of the medical and more practical engineering fields.

**Figure 3:**
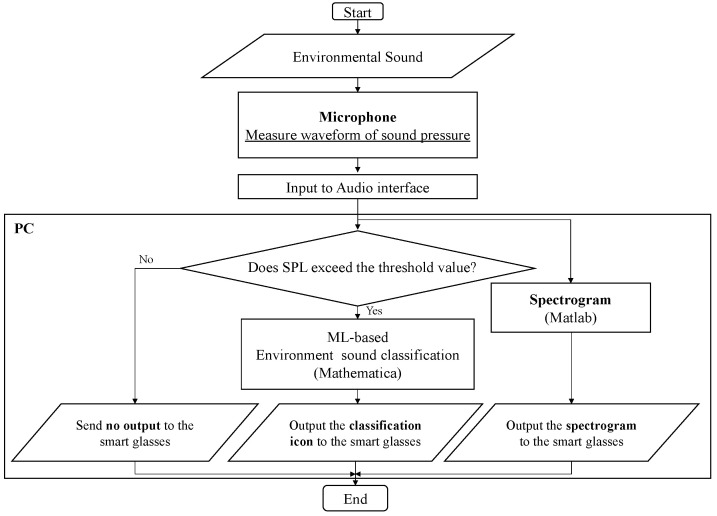
Flowchart of the visual presentation system, including the signal processing of ML-based classification and time-frequency analysis to output the spectrogram.

The process flow for recognizing environmental sounds and displaying icons and spectrograms is shown in Figure 3. First, environmental sounds generated in the surroundings are continuously detected by a microphone. The sound pressure waveform detected by the omnidirectional microphone attached to a sound level meter (Rion, NL-62) is output from the sound level meter and input to a laptop PC for control via an audio interface (Steinberg, UR22mkII, Hamburg, Germany). Then, the sound pressure waveforms are classified by the ML-based classifier and converted into a spectrogram. Herein, the conversion of sound waves into the spectrogram was conducted using the Spectrum Analyzer of Matlab. The setting of the analyzer was as follows: a sampling frequency of 48 kHz, a method of “Filter bank” [67], a frequency range of 70 Hz to 7 kHz, a time duration of 9 s, a unit of dBm/Hz, and a logarithmic frequency scale. By setting these parameters, the time–frequency characteristics of the sound waveforms of the newest 9 s were continuously displayed.

The sound level meter is inserted in the side pocket of a backpack so that the microphone part protrudes from the backpack and can appropriately capture nearby environmental sounds. As shown in the flowchart, when the sound pressure waveform captured by the microphone is input into the processing system, the system first determines whether the sound pressure level exceeds the pre-measured sound pressure level of the background noise in the target room. The in situ background noise level was measured by a sound level meter prior to the experiment on each experimental day, and the measured value of the level was input into the software and used as the threshold value of the background noise. In the residential space where the experiment was conducted, there was a concern that some icons would be displayed even though no household sounds were heard, due to fluctuations of the sound pressure level inside the room caused by such external noise as vehicles running. For this reason, time-series data on the sound pressure level in the experimental room during the daytime hours were obtained in advance, and the standard deviation for the sound pressure level was calculated, which tended to converge around 2 dB. Therefore, taking into account this fluctuation, a threshold sound pressure level was set to the value of 5 dB plus the sound pressure level averaged over 30 s, when no vehicle running noise was heard. When the sound pressure level exceeds the threshold value set as above, the measured sound pressure waveform is input into the classifier generated by ML in advance, and the type of environmental sound is determined. When the sound pressure level is below the threshold value, the environmental sound is not classified, and no icon is displayed. Thus, if the user is alone in the house and it is very quiet, then only a spectrogram without any sound icons is displayed on the screen. In the processing system, the ML classification results are updated every 3 s. This is because almost all single-occurrence sounds are rarely shorter than 3 s, considering the reverberation in the room where they occur. The intention was to increase the time interval between updates of the icons as much as possible, because it has been noted [35] that when visualizing information of environmental sounds on a display, frequent changes can be distracting, and users seek visual silence. However, because the spectrogram of the acoustic signal over 9 s is always displayed, the user can check the spectral characteristics up to 9 s ago. This time length was also chosen to reduce the stress on the user as much as possible as described above while displaying for as long a time as possible, and conversely to ensure that the notation of the spectrogram of the sound would not become too small or difficult to understand by being too long.

### 3.3. Limitations of Proposed System

The current system is limited to the most prominent sounds generated inside a house, and the display system depends on the results of the ML classification. Although it is necessary to recognize the sounds of the external environment, the background noise in external environments is prominent and real-time ESR by ML is currently still difficult [43]. In addition, it is difficult to conduct onsite outside experiments that are safe for subjects, so this study was limited to household interior sounds. However, in view of the final results of this study, it is considered to have the potential to be applied to outdoor ESR in the future.

From a software perspective, the system in this study used a Mathematica-based classifier for environmental sound recognition, while the dynamic spectrogram was displayed by Matlab. In terms of integration and extensibility, it is preferable to unify the operation on python or Matlab, but in this study, the environmental sound detection was first examined using the function on Mathematica. So, in order to make use of the learned classifier as it was, a separate Matlab-based spectrum analyzer function was additionally used to display the dynamic spectrogram. In the future, it will be efficient to operate the system uniformly on a single software package, and as described below, the selection of software is also a key factor when looking at the generalization of the system.

In this study, the visual contents converted from auditory information were displayed using see-through AR glasses. Since these are output through software processing, it may cause delays due to the processing and may induce cybersickness, which has recently become apparent [68]. In the present study, the subjects were taught to immediately verbally request that the experiment be stopped immediately if they had symptoms of VR sickness. However, since no subject offered to do so, it was assumed that VR delays that would make them feel sick did not occur in the least. However, such factors that undermine user convenience should be eliminated, and considering that the processing system will be moved online or that the processing unit will be embedded in the glass itself, it is necessary to continuously examine the impact of processing system delays. Furthermore, in the future, when considering the lightweighting and generalization of the system, it will be necessary to additionally verify to what extent the performance of the processor can be reduced and still be used within the range of delays that are practically available.

Household sounds, such as the sound of running water in a distant kitchen, have very low sound pressure levels, and show almost the same level of sound pressure as background noise, but their low sound pressure levels do not mean that they are less important. In the present study, the sound pressure level was set to the level of the background noise plus 5 dB, which eliminated the possibility that meaningless background noise would be recognized as an environmental sound. On the contrary, there were some cases where sounds with very low sound pressure levels were not recognized. Such misleading results may be improved by considering not only the threshold determination of the sound pressure level, but also the spectrogram of the background sound. However, while such an improvement in accuracy is possible, the computational load increases, so it should be an issue for the future.

Because converting auditory information into other modalities and displaying it can support DHH people, it is not necessary to limit the means of conversion of environmental sounds to visual information. For example, Yağanoğlu and Köse [17] presented environmental sounds to DHH people by means of vibrations, and their effectiveness was verified. However, it is also considered that the amount of information that can be presented is limited to the modal vibration alone, but such a system can be helpful for someone who is also visually impaired. Moreover, AR devices are becoming gradually lighter [69] and are expected to become increasingly convenient in the future. Furthermore, previous studies have pointed out that in sound visualization systems for DHH people, the type and arrangement of the display are very important [42], suggesting that it is important to place the information in an easily viewed position. With this in mind, the present study adopted smart glasses to present visual information so that the sound information was always visible. However, the possibility of more intuitive ESR by additionally using vibration information is also expected as a future work.

On the other hand, there have already been many attempts to visualize sound, such as visualization using icons and spectrograms [35], or visualization methods using gestures [50], which can make it easy to grasp sound intuitively. However, the present study adopted only icons and spectrograms and investigated the effectiveness of displaying both of them simultaneously on smart glasses. The current system did not focus on intuitive comprehension; therefore, in terms of intuitive understanding and comprehensibility, the findings of the current study should be developed as a more user-friendly system in future work.

## 4. ML-Based Environmental Sound Classifier

The flow of the current section regarding the generation and validation of the ML-based environmental sound classifier is shown in Figure 1. First, as shown in the flow, the detailed measurement methods of the data sets and the acoustic properties of the measured sound data are described in Section 4.1. Then, the creation of the ML-based classifier is described in Section 4.2. Finally, the validation results of the practical classification performance of the resulting classifier are described in Section 4.3. The details of all contents are described as follows.

### 4.1. Measurement of Training Data

Environmental sounds of daily life were recorded to obtain training data for use in supervised ML. Specifically, the 12 kinds of environmental sounds shown in Table 2, which can be detected in the household space of daily life, were adopted following the survey results of Section 2. From the suggested issues related to the interior space, specific keywords related to events that DHH people have trouble hearing are extracted as follows: sounds of alarm, cooking, running water, washing machine, vacuum cleaner, raining, knocking on door, dropping something, intercom (doorbell), human voice including baby crying, and footsteps. The cooking and washing sounds are examined in Experiment II of Section 5 because the timing of their finishing operations was considered an alarm sound in the current study. So, among the above items, the sounds of alarm, intercom, human voice, knocking on a door, and footsteps were adopted. In addition, although at a weak sound pressure level, sounds related to the opening and closing of doors are important information, as well as the sound of knocking on doors, and previous studies have also commented on events where doors are opened and people approach [42]. Additionally, as for the sound of dropping something, the sound of dropping a plastic bottle was used as an example of a relatively clear sound, while the sound of dropping a purse was an example of a sound that is relatively difficult to notice. It should be noted that, although the sounds of an alarm and intercom are artificial electronic sounds and have similar acoustic characteristics, their roles are different, such as the sound that announces the completion of some home events and the sound that announces a visitor. Previous studies [42] have also distinguished between doorbells and alarms. For these reasons, these sounds were distinguished in this study. The above sounds were intentionally generated, and the sounds were recorded at a sampling rate of 48,000 Hz using an omnidirectional microphone (Rion, NL-62) and an audio interface (Steinberg, UR22mkII). The generation and validation of classifier as well as the computational procedure of ESR in the validation experiments of Section 5 were processed using a note PC with CPU and GPU (Mouse Computer, Tokyo, Japan, NEXTGEAR-NOTE i5730SA1) as follows. The CPU was Core i7-7700HZ (2.8 GHz). The GPU was GeForce GTX 1070 (8 GB GDDR5). The installed memory and HDD were 16 GB and 256 GB SSSD, respectively.

Among the 12 kinds of sounds, 10 of them, excluding male and female human voices, were recorded in three different residences identified as rooms A, B, and C in Table 3, while the human voices were measured inside an anechoic room. Detailed information on these measurement rooms is shown in Table 3. The rooms where the target environmental sounds were generated were 33.5 m^2^, 9.1 m^2^, 30.0 m^2^, and 3.8 m^2^. Each of these rooms was located in different houses with wooden or reinforced-concrete structures. To increase the prediction accuracy, environmental sounds were produced and measured inside rooms with different sizes, furniture arrangements, door and floor materials, and acoustic conditions.

When recording these various environmental sounds, the waveforms measured using common alarms, plastic bottles, purses, and pedestrians in each of rooms A, B, and C were used for the sounds of alarms, dropping plastic bottles and purses, and footsteps, while the other sounds were generated by different intercoms in each house or by running water into sinks made of different materials in each house. For the voices, one male and one female in their 20s were selected and the five kinds of Japanese vowels (/a/, /i/, /u/, /e/, and /o/) uttered by them were adopted as the sound data of voices.

**Figure 4:**
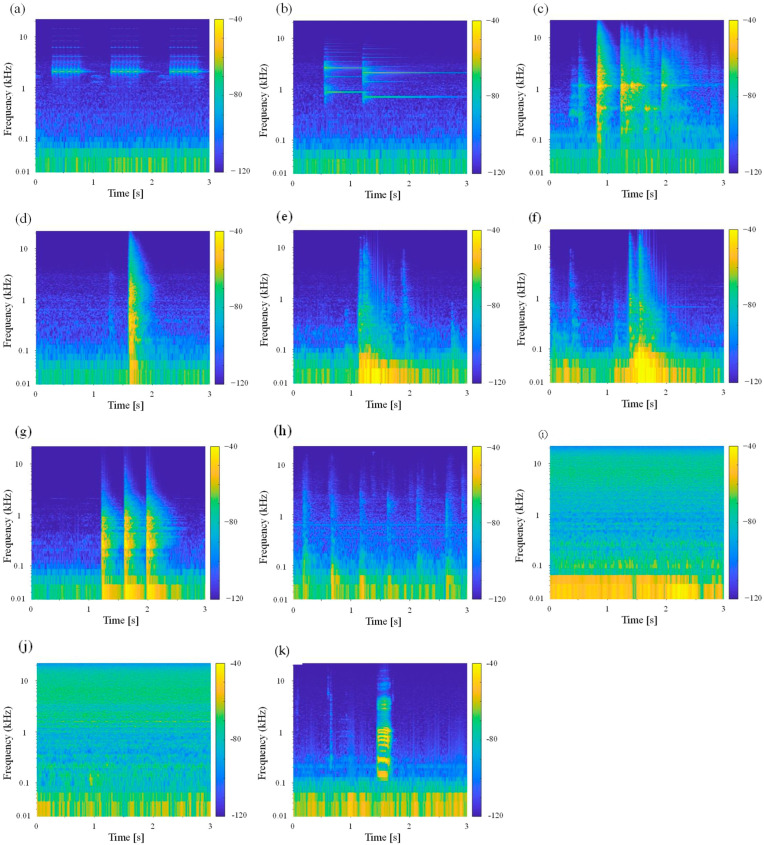
Spectrograms of each of the 12 measured sounds; (**a**) alarm, (**b**) intercom, (**c**) dropping plastic bottle, (**d**) dropping purse, (**e**) opening door, (**f**) closing door, (**g**) knocking on door, (**h**) footsteps, (**i**) running water from the sink, (**j**) running water from the washbasin, and (**k**) human voice, respectively.

To consider the characteristics of the sound data, spectrograms of the measured environmental sounds are shown in Figure 4. Note that only the male voice is indicated in this figure because the male and female voices had similar spectrograms. In these spectrograms, as an example, all environmental sounds other than speech are shown as waveforms measured in room C, while the speech data show the sound of the vowel /a/ pronounced and recorded in the anechoic room. First, as the alarm and intercom sounds consist of several pure tones superimposed on each other, characteristic peaks at the frequencies of these pure tones can be seen. Although this feature is considered to be more easily recognizable than other environmental sounds, the similarity of the frequency characteristics of the alarm and intercom sounds may raise concerns about misidentification between alarm and intercom sounds.

Next, the characteristics of the transient environmental sounds of dropping a plastic bottle, dropping a purse, a door opening and closing, knocking on the door, and footsteps are described. Note that the plastic bottle and the knocking sound were recorded as sounds with several transient sounds continuously occurring due to several bounces of the dropped object or three knocks. In terms of the frequency response, these pulsive sounds have a broadband frequency component that differs from a pure tone. Additionally, for these sounds, the pulsive sounds occur first, after which the remaining sound decays. As the average sound absorption coefficient in the measured room increases at higher frequencies, the frequency response of the reverberation time is basically shorter when the frequency is higher. As a result, all transient sounds tend to decay more quickly at higher frequencies. The sounds of plastic bottles and purses dropping and knocking on doors are similar in that they have a wide range of frequency components and may be misinterpreted. In addition, the sound of knocking on doors could also be recognized in terms of such periodicity, as they were learned as a knocking sound repeated three times. Although the sounds of a door opening or closing show audible differences, there are not many differences in the spectrograms, so it may be difficult to classify these sounds with ML. Second, each flowing sound is a steady sound and has a broadband frequency component, which is similar to the silent state and may be difficult to distinguish. However, in the present study, if the sound pressure level in the field is below the threshold sound pressure level of the background noise, then the sound is not classified using ML results, so it can be distinguished from silence. Finally, it is assumed that the spectrogram of a voice is relatively easy to distinguish from other environmental sounds, as it has discrete formant frequencies that are represented as stripe patterns, which is quite different from the frequency response of other environmental sounds.

### 4.2. Generation of Classifier of Environmental Sound by Supervised ML

**Figure 5:**
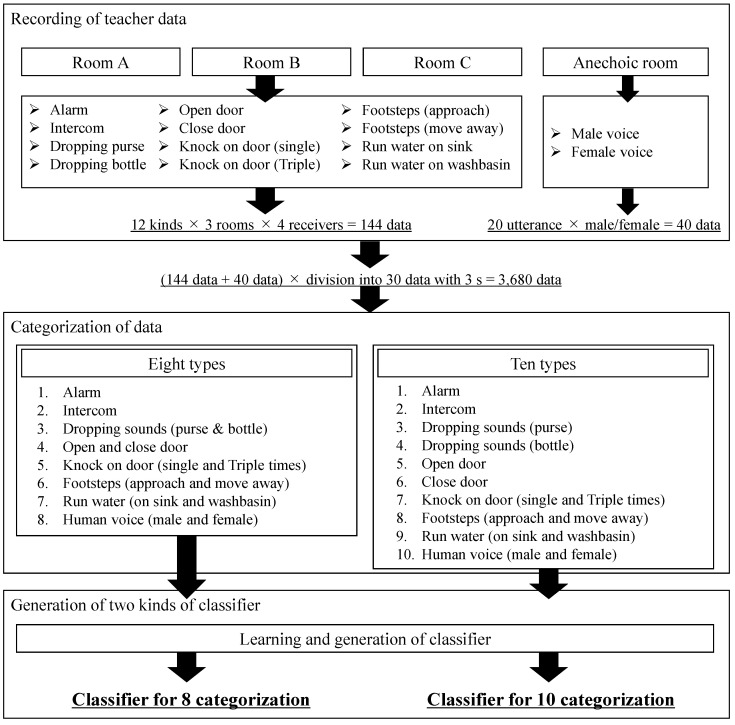
Flowchart of generation of classifier.

To classify each environmental sound using ML, the classify function in Mathematica from Wolfram Research was employed to perform supervised learning using the obtained sound pressure waveforms. In supervised learning with Mathematica, the environmental sound data are first converted into a mel-frequency spectrum, a multi-dimensional vector is generated, and general processes such as normalization and dimensionality reduction are applied to prepare the training data, which are then trained by a neural network. The procedure indicated in Figure 5 and the following procedure describes the method to create training data as the input data for this function.

1. Sound data for 5 s of each environmental sound were recorded in each of rooms A, B, and C. Here, the sounds of alarm, intercom, dropping a plastic bottle and purse, door opening and closing, and running water in the kitchen sink and washbasin were recorded as four kinds of sound data obtained at four different receiving points. The sounds of footsteps and knocking on a door were recorded as eight kinds of sound data, including two kinds of knocking patterns (single or three times) and two kinds of footsteps (approaching or moving away from the receiver) obtained at four different receiving points. In total, 144 sound data sequences were obtained in the three rooms.
2. For the purpose of watering down the sound data, 20 kinds of sound data with a time duration of 3 s were extracted from each of the original sound data sequences by changing the starting point of the extraction in 0.1 s increments.
3. By carrying out the process described in step 2 for all of the measured environmental sounds, a total of 144 × 20 = 2880 sets of data were created. However, as the sound data of human voices were measured only in an anechoic room, 40 kinds of voice data, including 20 data sets for each of male and female voices, resulting in 40 × 20 = 800 sets of training data, were prepared and used as the training data for step 1.
4. As a result of following the above steps, 2880 + 800 = 3680 sets of training data were obtained. Using these data, a classifier was generated, and the accuracy of classification with this classifier was verified. It should be noted that there is room for considering whether excessively detailed ESR information should be presented to DHH people. For example, sufficient consideration needs to be given to whether the sound of running water in a sink or a washbasin should be classified and learned as separate categories or as the same category of “running water sound”. From the viewpoint of classifier generation, environmental sounds with similar characteristics are difficult to distinguish from each other, so the balance between what kind of information a DHH person wants to know and how accurately the ML-based classifier can classify the sounds are also important issues. Furthermore, how to set up the categorization of environmental sounds in ML is also important, as the misclassification of environmental sounds was indicated as a problem in a previous study [42]. Based on these considerations, the present study compared and evaluated each case in which a total of 12 types of sounds, including the 10 environmental sounds and 2 male and 2 female voices described above, were learned by categorizing them into 10 or 8 types, as shown in Table 4. Note that, as described in Section 2, if the sound pressure level was below the threshold sound pressure level, then it was judged to be silent and not applied to the classifier; if the sound pressure level was greater than the threshold level, then it was judged to be an environmental sound occurrence and applied to the classifier.

### 4.3. Validation Experiment for ML-Based Classifier

#### 4.3.1. Method

**Figure 6:**
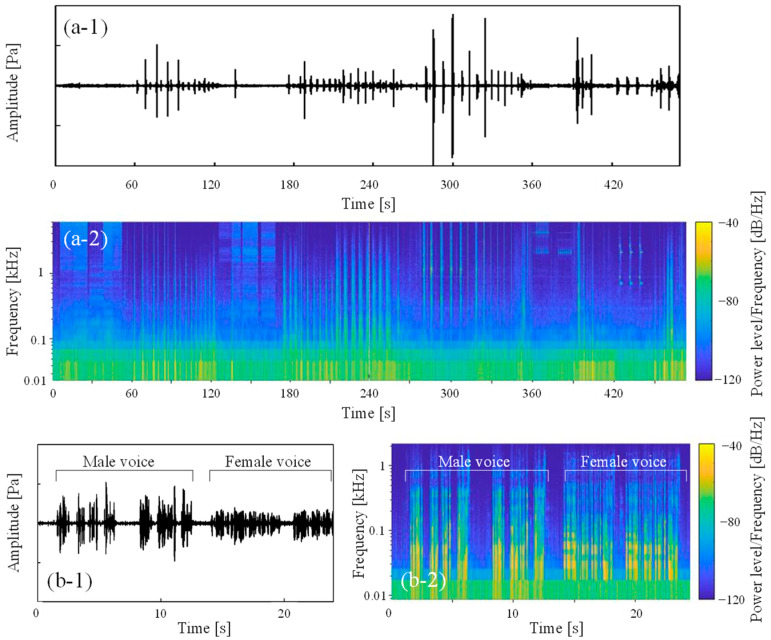
Time waveform and time–frequency spectrogram of (**a**-**1**,**a**-**2**) environmental sounds other than human voices and (**b**-**1**,**b**-**2**) human voices.

Two types of continuous sound data were recorded to investigate the feasibility of classifying environmental sounds in dwellings by ML. The first type of sound data was recorded by intentionally and intermittently generating sounds other than the human voice. This recording was carried out in room C, where the subjective evaluation experiment described in the next section was conducted. The second type of sound data was recorded by having a male and a female speak in an anechoic room. In the latter voice recording, each of the male and female subjects was asked to utter a line of about 6 s each in Japanese to introduce themselves with their name and the faculty they belonged to. As in the previous section, a sound level meter (Rion, NL-62) with an omnidirectional microphone, a USB audio interface (Steinberg, UR22mkII), and a laptop PC were used for recording. In this recording, the environmental sounds were generated as randomly as possible. As a result, two kinds of sound data with a duration of 417 s for environmental sounds in room C and male and female speech data with a duration of 24 s were obtained. The measured waveforms and spectrograms are shown in Figure 6. The non-voice environmental sounds were then split into 157 segments of sound data 3 s-long, and the latter voice data into 8 speech data segments also 3 s-long.

#### 4.3.2. Evaluation of Classification Results

The classification results can be expressed as a confusion matrix. The structure of the confusion matrix is indicated in Table 5. In this table, the rows represent the true classes of the samples, while the columns represent the predicted classes. Herein, to evaluate the accuracy of the estimated results, we adopted the following four kinds of indices: accuracy, recall, precision, and *F-score* calculated using *TP*, *TN*, *FP*, and *FN*, as indicated in the table. Because the recall and precision have a trade-off relationship, the *F-score*, which is the harmonic mean of recall and precision, is also used, as in Equation (4): the closer the F-value is to 1, the more balanced and higher the performance is.

(1) $$Accuracy=\frac{TP+TN}{TP+TN+FP+FN},$$

(2) $$Recall=\frac{TP}{TP+FN},$$

(3) $$Precision=\frac{TP}{TP+FP},$$

(4) $$\mathrm{F-score}=\frac{2\times Recall\times Precision}{Recall+Precision}$$

#### 4.3.3. Recognition Results and Discussion

**Figure 7:**
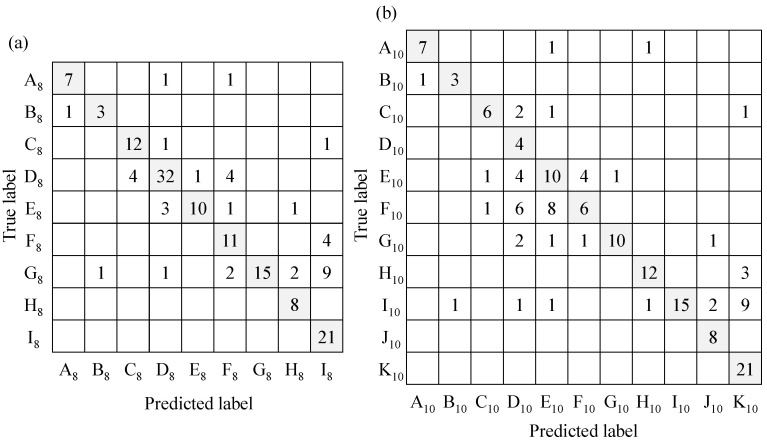
Confusion matrix of each condition of classification into 8 or 10 sub-categories. The labels A_8_ to J_8_ in (**a**) and A_10_ to K_10_ in (**b**) are defined in Table 6**.** The shaded parts indicate the number of accurate classification.

The 157 environmental sounds and 8 speech data sets were classified by an ML-based classifier, and the results were evaluated using the above methods. The evaluated results of the classifier with 10 or 8 sub-categories are shown in Figure 7 as a confusion matrix and Table 6 as the various indices.

The confusion matrix of the classification results and each index are compared between respective categorizations. First, Table 6 shows that the classification accuracy was higher in the condition of categorization with 8 types than that with 10 types. The number of misclassifications was reduced by grouping the dropping sounds and the door opening/closing sound together as one category for each set. However, the confusion matrix showed that there were many misclassifications within transient sound categories, even with 8 sub-categories, so it is expected to be effective for users to compensate for this by visually interpreting the spectrograms. Although the condition with 8 sub-categories indicated higher accuracy, not all of the F-scores of the condition indicated higher values. For the sounds of knocking on the door and footsteps, the F-scores were higher in the condition with 10 types of identification than that with 8 types. For these sounds, it can be considered that the combination of the dropping sound and the door opening/closing sound conversely increased misclassification, and the difficulty of sound recognition within the transient sound category is once again confirmed. However, when assuming the use of the system in daily life, for example, if the classification result is door opening/closing when the user sees their cohabitant in front of them, then the user can judge that it is a misrecognition because there is no other person in the room. In actual daily use of the device, appropriately incorporating the user’s judgement in such situations would be important. The rate of misclassification was low for tonal sounds such as alarms, intercom tones, and voices. In addition, there were few errors among these tonal sounds, and it was possible to distinguish between alarm sounds consisting of intermittent tones of the same frequency component and intercom tones consisting of compound tones with different frequencies without misidentification. Second, the F-score of the sound of running water was low, as shown in Figure 7. Here, a large number of these sounds were recognized as silence because the washbasin and the sound receiving point were far apart and the sound pressure level of the running water sound was attenuated by distance, which meant that the sound pressure level in many conditions did not exceed the threshold level. However, if the threshold of the sound pressure level for the silent state is lowered any further, then the ML system will display an incorrect recognition result, even when the human perceives the sound as silent, which may cause stress for the user. In such a situation, it is expected to be effective to compensate for these situations by visually observing the real-time spectrogram. From this discussion, it was judged that ESR with the ML-based classifier with 8 sub-categories was more effective than that with 10 sub-categories. Therefore, the experiments described in the next section were conducted with the support and ML-based classifiers assigning sounds to 8 sub-categories.

## 5. Evaluation of the Proposed System

The results presented in the previous section show that the detection of environmental sounds by ML had a recognition accuracy of around 70% for a classifier with eight sub-categories, and that even in this condition there were some sub-categories for which the recognition accuracy was not still perfect. Therefore, to complement the recognition accuracy by ML, the presentation system proposed in this paper also illustrates the visualization results of the time–frequency characteristics of environmental sounds by means of spectrograms. As mentioned above, training has been shown to improve the ability to recognize speech with spectrograms. Greene et al. [48] showed the results of 2 months of training, while Farani et al. [49] showed that recognition ability increased after only 10 training sessions. However, this training was limited to speech recognition. Therefore, this study also confirmed that training improved the ability to recognize environmental sounds. As shown below, first, the effectiveness of the training at enabling users to understand what the spectrograms showed was verified by Experiment I. Subsequently, the effectiveness of our presentation system was verified by Experiment II. In this experiment, normal-hearing people who simulated hearing impairment by wearing earmuffs were employed as subjects. The experiment was designed to identify the types of household sounds that occur intermittently in the house and to have the subjects perform required daily tasks that are related to the environmental sounds. Details of Experiments I and II are described below.

### 5.1. Experiment I

This experiment was conducted to test the effect of training on the ability of subjects to identify the type of environmental sound after seeing the visualized spectrogram on the smart glasses.

#### 5.1.1. Method

The environmental sounds measured in the previous section were used as the task sounds. Specifically, subjects were asked to identify whether the spectrogram presented on the smart glasses was an alarm sound, intercom sound, dropping sound, sound of door opening or closing, sound of knocking on the door, footstep sound, sound of running water, or human voice. We used 15 subjects aged between 22 and 25 years. They were asked to perform three ESR tests based on the spectrogram. Between each test, training on sound identification using a spectrogram was also carried out. The detailed procedure is described below.

First, the procedure for creating the training content is described. Among the environmental sounds measured in Section 4, three different waveforms were prepared for each of the eight types of sounds described above, for a total of 24 sound source data sets. The duration of all environmental sounds was 5 s.

**Figure 8:**
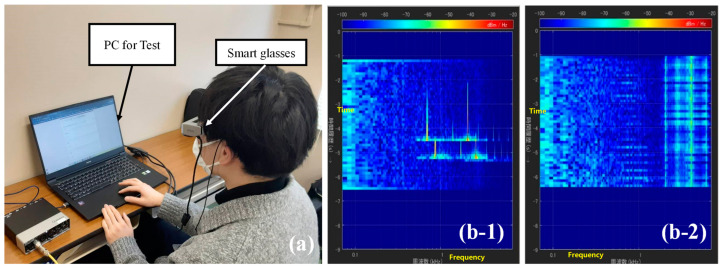
(**a**) Experimental setup of Experiment I and two examples of the spectrograms: (**b**-**1**) intercom sound and (**b**-**2**) alarm sound.

The training and experimental procedures are described next. In the training and experiment, the spectrogram was viewed on the same smart glasses as used in Experiment II, as shown in Figure 8. As shown in the figure, the real-time display of the spectrogram can be viewed up to 9 s before the current time, with the time on the vertical axis set to 9 s. In the study, basic information about the spectrogram was first explained to the subject. Specifically, the subject was taught that the vertical axis represents time (in seconds), the horizontal axis represents frequency (in hertz), and the colored bars represent sound pressure (in decibels). The subjects were shown how each of the sound events is displayed as flowing in the vertical direction. Note that the spectrogram can be displayed in amplitude or in decibels, but because previous studies [51] have shown that the decibel notation of amplitude makes it is easier to interpret the sound visually, the amplitude was displayed in decibels in this study. The experimental procedure is described below.

1. Each type of environmental sound was shown to the subjects while they watched the spectrogram flowing in real time. On the spectrogram, a 5 s waveform prepared as described above was shown flowing on the time axis from top to bottom. Subjects could ask to see the 5 s spectrogram again.
2. The subjects took the first test comprising 24 questions. In the test, the subjects wore smart glasses as in Figure 8a, and the spectrogram was dynamically displayed on the smart glasses. Note that, although the spectrogram could be viewed again during the pre-study, in all three main tests, each spectrogram could be viewed only once.
3. After the first test, the spectrogram was again displayed in real time according to the procedure described in step 1, and the subjects were asked to identify the environmental sound while being shown each spectrogram.
4. The subjects took the second test, following the same procedure as in step 2.
5. The subjects took the third instruction session using the same procedure as in step 1.
6. The subjects took the third test, following the same procedure as in step 2.

The above six steps were carried out by each subject. Note that the three main tests were conducted on different days.

#### 5.1.2. Results

**Figure 9:**
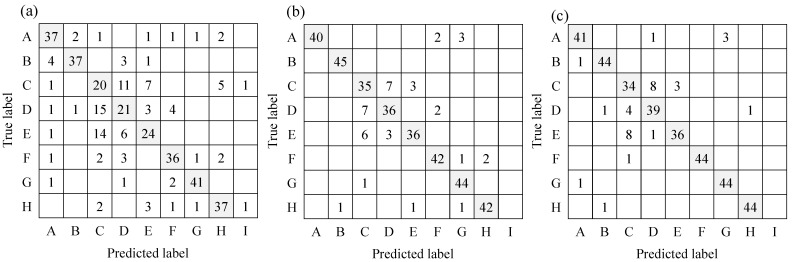
Confusion matrix of each of the conditions for the (**a**) first, (**b**) second, and (**c**) third tests. The sound labels indicate A, alarm; B, intercom; C, dropping object; D, door opening/closing; E, knocking on the door; F, footsteps; G, running water; H, human voice; and I, not identified. The shaded parts indicate the number of accurate identification.

The results of the three tests are shown as confusion matrixes in Figure 9 and the evaluated indices in Table 7. Table 7 shows increased accuracy approaching 1.0 in the three trials, indicating that subjects were trained to be able to identify environmental sounds from the spectrogram. Particularly, the accuracy for the third test was as high as 90%, indicating that ESR during implementation in daily life is likely. Next, it can be seen that the F-scores of all the environmental sounds increased in the second and third trials. The F-scores of the tonal sounds, such as alarm, intercom, and human voice, exceeded 90%, indicating that they were easy to identify from their visually distinguishable patterns. Transient sounds such as dropping sounds, sounds of opening/closing doors, and knocking on doors had a smaller F-score than those of the other categories. As can be seen from the example spectrograms of Figure 4, the diagrams of the transient sounds are very similar, indicating that they were difficult to distinguish from each other. However, the confusion matrix shows that within the same transient sound category, for example, a dropping sound was often misidentified as a door opening or closing sound, indicating that the system was able to recognize that some sudden sound was occurring even if it was not correct enough to identify the detailed sound information. For footsteps, the ML-based results often showed them as silent and produced low F-scores, whereas the present experiment, based on identification by spectrogram, showed relatively high values. As the transient sounds were limited to low frequencies, the frequency characteristics were concentrated more on the left side of the spectrogram, with a short period of about 1 s, and the characteristics of the periodic occurrence made the identification of footstep sounds relatively easy. The same discussion as above can be applied to the sound of running water, with a high F-value. A benefit from supplementing the ML results with the spectrogram appearance is expected.

Thus, even if it is not possible to precisely recognize detailed information about a sound, it is at least possible to identify whether the sound is transient or stationary, and whether it is electronic. This is considered useful in recognizing events occurring in situ in everyday life. The validity of the complementarity between them is discussed in the next section.

### 5.2. Experiment II

In this experiment, the effectiveness of the proposed system in daily life was evaluated by asking subjects to rate the subjective impressions they perceived when using the system. The ML-based classification results and real-time display of the spectrogram described in the previous sections were presented on smart glasses to study the possibility of performing tasks prompted by changes in the surrounding environment.

#### 5.2.1. Method of Experiment

The aim of this experiment was to verify whether normal-hearing people can perform tasks of daily life in a condition that simulates hearing impairment, become aware of environmental sounds, and perform the tasks associated with these sounds. This experiment was conducted in room C, as described in Section 3 and Table 3.

The experiment was conducted with 15 subjects aged 22–25 years, all of whom had studied the spectrograms in the previous section. The following two types of experiments under different conditions were conducted:

1. Exp-II-Ic: Only the ML-based icons were presented on the smart glasses.
2. Exp-II-IcSp: A real-time display of the spectrograms and the ML-based icons were presented on the smart glasses.

**Figure 10:**
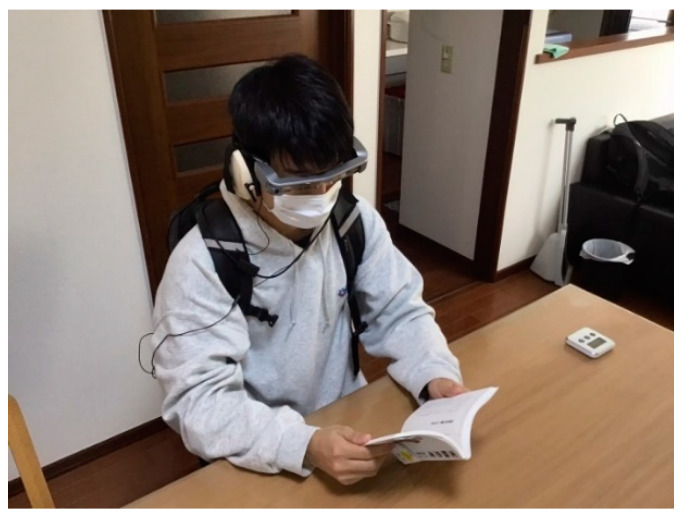
State of a subject with all the experimental equipment attached, performing the standby task of reading during the experiment.

We compared the results of the above two conditions. Subjects wore the following devices to intentionally create a situation where they would be unlikely to hear nearby sounds. First, dynamic insert earphones (Etymotic Research, MK5, Elk Grove Village, IL, USA) were worn, from which white noise was reproduced to mask ambient sounds. With the earphones inserted into the ear, earmuffs (3M, Peltor H6B/V, Saint Paul, MN, USA) were worn over the earphones to block the propagation of ambient sound into the ear. The sound insulation performance of the earmuffs was 21 dB. Finally, smart glasses were worn to present visual information. Note that the subjects were asked to carry a backpack containing a laptop PC for processing the information to be displayed on the smart glasses, an omnidirectional microphone (Rion, NL-62, Tokyo, Japan) for real-time sound measurement, and an audio interface (Steinberg, UR22mkII) for analog/digital conversion. A subject with all of the above devices attached performing the standby task of reading during the experiment is shown in Figure 10. Here, the ML-based icons and the spectrograms are visible on the smart glasses as shown in Figure 2. The icon is updated every 3 s. In the real-time display of the spectrogram, the time on the vertical axis is set to 9 s, as in the training conducted in the previous section, and the history from the present to 9 s ago can be checked by the user. Note that Exp-II-Ic and Exp-II-IcSp were conducted on different days, with Exp-II-IcSp conducted earlier than Exp-II-Ic. As the two tests were conducted on different days, the experience of the first experiment was considered to have a minor influence on the results of the second experiment, but just in case there was a possibility of habituation of the system and a learning effect on the usage of this system, Exp-II-IcSp, which was expected to produce relatively high ESR scores by the subjects due to the relatively large amount of information provided by combining spectrograms and icons, was conducted first. In addition, to ensure that the learning effect of the spectrogram did not decrease, the experimental dates were set so that Experiment I and Exp-II-IcSp were conducted within 1 week of each other.

#### 5.2.2. Experimental Daily Tasks

This experiment investigated how the accuracy of ESR changes when only ML is used as a criterion and when both ML and spectrograms are used as criteria, and how the ease of performing the household tasks was affected by these changes.

First, to verify the accuracy of ESR, during the experiment, subjects were asked to verbally say the identification result of a sound that was generated at the time, based on the information presented by the smart glasses. Furthermore, if there is a task associated with the identified sound, then the subject was also asked to say the task out loud as well. Note that when this experiment was conducted, only two persons were present inside room C (the subject and the person in charge of the experiment) to control the experimental conditions. The person in charge of the experiment deliberately generated various environmental sounds around each subject. The subjects were instructed to assume that this person in charge of the experiment was his or her roommate. When the person in charge generated the environmental sounds, he took care to ensure that the sounds were generated at a position out of sight of the subject so that the subject could not recognize the generation of the sounds by sight. Then, the person in charge judged the correctness of the identification results and created a confusion matrix. The duration of the experiment was set at around 20 min per session to avoid subject fatigue.

The tasks to be carried out after the recognition of the environmental sounds are described below. Subjects were instructed in advance to perform the following tasks using a task list kept at hand so that they could immediately reconfirm the tasks.

1. Set an alarm for the washing machine.

The subject loaded towels into a washing machine and set an alarm for 15 min. The alarm was attached to the washing machine, and the subject was expected to recognize the finishing time by the alarm sound.

2. Set an alarm to boil eggs.

Eggs were placed in a pan of water that was then placed on a heated stove until the water started to boil. After the water started boiling, an alarm was set and the eggs were boiled for 9 min over medium heat. The 9 min were measured by a different alarm from the one used in the washing machine task. When the sound of the alarm was detected, the subject turned off the heat and peeled the shells off the eggs.

3. Interact with delivery staff.

During the experiment, an intercom was activated by the person in charge. Subjects recognized the sound and picked up the intercom receiver to identify the visitor.

4. Identify a dropped object.

An object was dropped by the person in charge. Subjects recognized the sound of something dropping and looked for the dropped object.

5. Open the door in response to a housemate’s knock.

The person in charge knocked on the door. Subjects recognized the knocking sound and opened the door.

6. Detect the end of the experiment.

The person in charge activated an alarm at the end of the experiment. The subject recognized the sound and deactivated the alarm.

In addition, subjects were asked to read a book as a standby task while they had nothing to do (e.g., while waiting for events and activities such as washing and cooking). As described in the questionnaire below, subjects were also asked to evaluate whether they could perceive whether the person in charge was in the room based on the information about the opening and closing of doors, walking in the room, and vocalizations.

**Figure 11:**
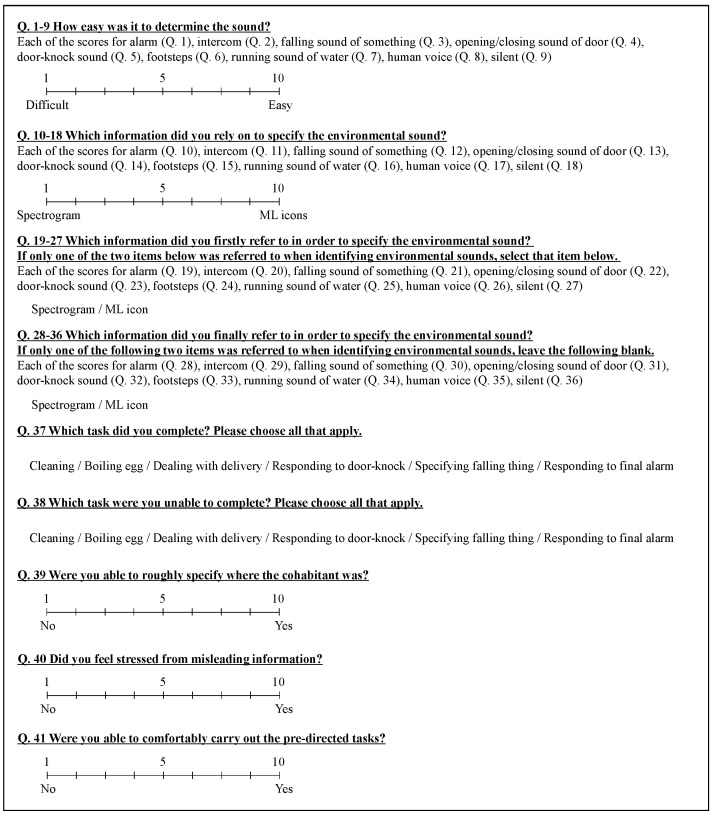
Questionnaire including 41 items used in the subjective evaluation.

After the experiment, the subjects were asked to complete a questionnaire for subjective evaluation. The 41 items included in the questionnaire are shown in Figure 11.

#### 5.2.3. Results and Discussion

**Figure 12:**
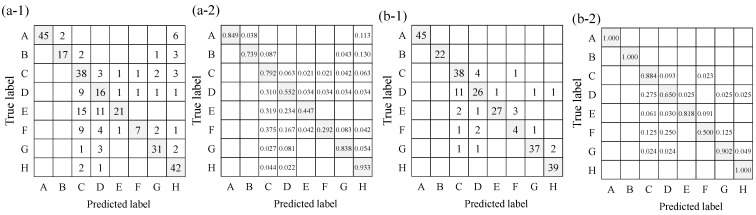
Confusion matrixes for the recognized results of environmental sounds in experiments (**a**-**1**,**a**-**2**) Exp-II-Ic and (**b**-**1**,**b**-**2**) Exp-II-IcSp. Panels (**a**-**2**,**b**-**2**) indicate the recall indices of Exp-II-Ic and Exp-II-IcSp, respectively. The labels indicate the following sounds: A, alarm; B, intercom; C, dropping object; D, opening/closing door; E, knocking on door; F, footsteps; G, running water; and H, human voice. The shaded parts indicate the number of accurate recognition.

The values for the confusion matrix and the evaluation index for each experiment are shown in Figure 12 and Table 8, respectively. First, the identification results of Exp-II-Ic are discussed. As shown in Table 8, the tonal sounds, such as alarm, intercom, and human voice, showed a high recognition accuracy, similar to the ML results in the previous section. Their F-scores were also high. However, there were a few situations in which the alarm and intercom sounds were recognized as human voice, and the accuracy was expected to improve in Exp-II-IcSp. Similarly high F-scores were observed for the sound of running water. This may have been due to the fact that stationary sounds were less likely to occur in the dwellings, resulting in less misrecognition. The environmental sounds that were difficult to recognize in Exp-II-Ic were transient sounds such as footsteps and knocking on doors. As described in the previous section, the spectrograms of the transient sounds had characteristics similar to each other, causing much misrecognition of the transient sounds. In addition, the subject’s own footsteps were sometimes misrecognized, but there is a possibility [70] that these can be distinguished and removed based on the characteristics of the footstep sounds of individuals.

Next, the results of the identification of environmental sounds in Exp-II-IcSp are discussed. It can be seen from Figure 12 that misrecognition decreased compared to the results of Exp-II-Ic. As can be seen from Table 8, each index for all environmental sounds also improved over Exp-II-Ic, and the real-time display of the spectrogram played a role in assisting recognition. In particular, the F-score of the alarm and intercom sounds was 1, suggesting that they can be recognized without any problems in everyday life. In contrast, the lowest F-scores for Exp-II-IcSp were obtained for footsteps, as in Exp-II-Ic. The subject’s own footsteps were sometimes mixed with other environmental sounds, resulting in misrecognition. In addition, the values of each index were higher for transient sounds compared to those in Exp-II-Ic, but the index values were still lower compared to other environmental sounds under the same conditions, indicating that misrecognition of transient sounds was frequent. Overall, however, Exp-II-IcSp showed higher values for each of the indicators, showing that the recognition accuracy was higher when the real-time display of the spectrogram was added than when only the ML recognition results were presented.

**Figure 13:**
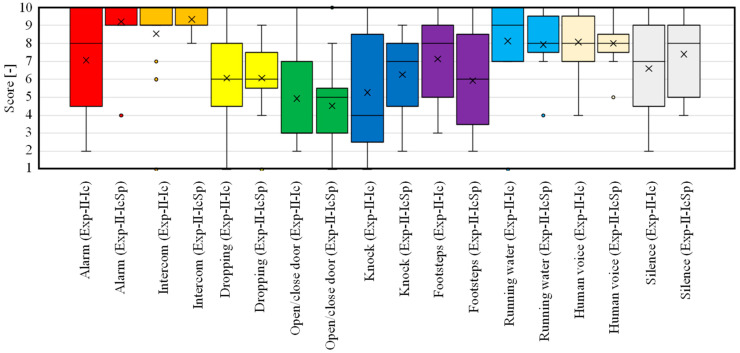
Results of subjective evaluation from questions 1 to 9, which represent the easiness of identifying each sound category. Larger (smaller) scores indicate that the subjects reported that it was easier (more difficult) to identify the sound. Outliers more than 1.5 interquartile ranges above the upper quartile (75%) or below the lower quartile (25%) are indicated as small circles.

Next, the results of questions 1 to 9 of the questionnaire are shown in Figure 13 to compare Exp-II-Ic and Exp-II-IcSp for each item. Student’s t-test was used to compare the two conditions, and a *p*-value under 0.05 is marked with an asterisk to indicate a significant difference between them. When Exp-II-Ic and Exp-II-IcSp were compared, the ease of identification was predominantly improved for the alarm sound, while the other categories did not indicate any significant difference in the ease of identification. Although the results of improved accuracy were obtained in Exp-II-IcSp, the subjective evaluation experiment used difficulty rather than accuracy as the axis of evaluation, so the increased difficulty of the spectrogram-reading task was evident, and resultantly, the difficulty level remained the same. In contrast, the environmental sound that significantly increased the difficulty of identification was footsteps. It can be seen from Table 8 that the precision decreased from Exp-II-Ic to Exp-II-IcSp, suggesting that other environmental sounds were recognized as footsteps, which increased the difficulty of recognition. This is a disadvantage of the increased information in the spectrogram.

**Figure 14:**
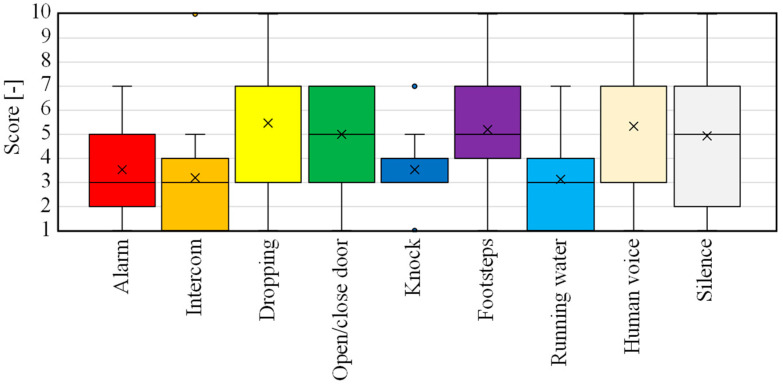
Results of subjective evaluation from questions 10 to 18, which represent the extent to which the subjects relied on the ML-based icons or spectrogram to recognize environmental sounds. Larger (smaller) scores indicate that the subjects relied more on the ML-based icons (spectrograms). Outliers more than 1.5 interquartile ranges above the upper quartile (75%) or below the lower quartile (25%) are indicated as small circles.

Next, the results of questions 10 to 18 are shown in Figure 14. This figure shows whether each environmental sound category was recognized by the ML-based icon or the spectrogram. The results indicate that for the alarm, intercom, door knocking, and running water sounds, the spectrogram was the main basis for identification. For the other environmental sounds, they did not rely on either one or the other but were considered to be referring to both of them.

**Figure 15:**
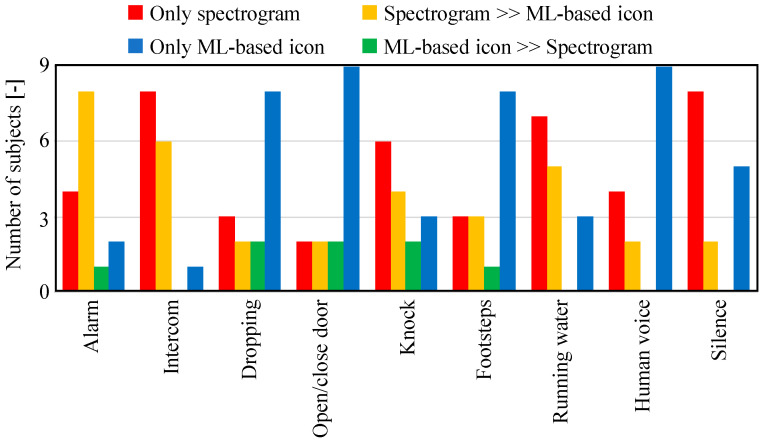
Results of subjective evaluation for questions 19 to 36. The distribution indicates whether subjects referred to each indication first (Q. 19–Q. 27) or last (Q. 28–Q. 36).

The results of questions 19 to 36 are shown in Figure 15. This figure shows whether ML-based icons and the spectrograms were used first or last when recognizing each environmental sound. These results indicate that many subjects referred only to the spectrogram or in the order of the spectrogram first and the ML-based icon last for the alarm, intercom, and running water sounds, which can be easily identified by the spectrogram. It is thought that many subjects identified the sound of door knocking from the spectrogram, because the door was knocked on three times. Thus, for sounds with clear frequency characteristics and periodicity, the spectrogram is considered able to easily identify their characteristics. In contrast, many subjects formed judgements only by the ML-based icons with regard to speech, which is thought to be due to the high accuracy of the ML classification. Moreover, many subjects referred only to the ML-based icons for dropping sounds, door opening/closing sounds, and footsteps, because recognition of these transient sounds by spectrogram is difficult. The results in the previous section also showed that the recognition of these transient sounds was difficult, which is attributed to misrecognition by ML.

**Figure 16:**
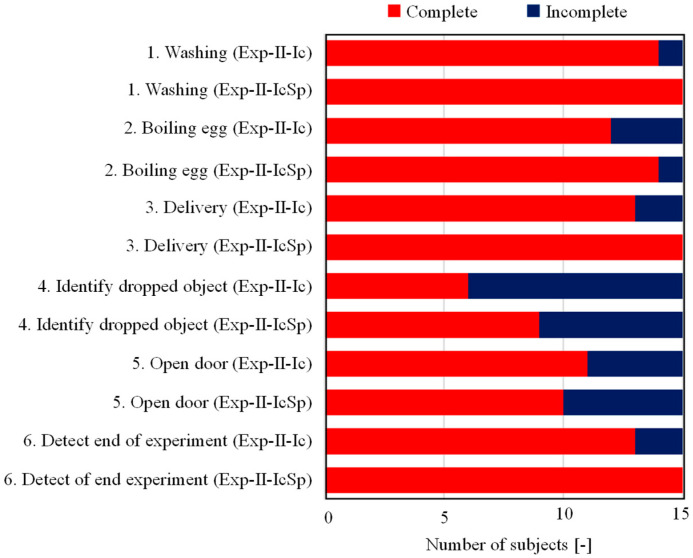
Results of subjective evaluation of questions 37 and 38. The distribution indicates the number of subjects who were able to complete (Q. 37) or not able to complete (Q. 38) the household task related to each of the recognized sounds.

The results of questions 37 and 38 are shown in Figure 16. First, it can be seen that more subjects were able to complete Exp-II-IcSp than Exp-II-Ic, with the exception of identifying dropped objects. In particular, all subjects were able to complete the tasks related to environmental sounds that were identified more accurately, such as washing clothes (alarm), responding to deliveries (intercom), and clearing the alarm at the end of the task. In terms of response to the sound of door knocking, more subjects were able to complete the task because they were able to recognize the sound. However, many subjects were still unable to complete tasks related to transient sounds, such as the identification of door knocking and dropped objects, and this was attributed to the fact that their identification accuracy was lower than that of other environmental sounds.

**Figure 17:**
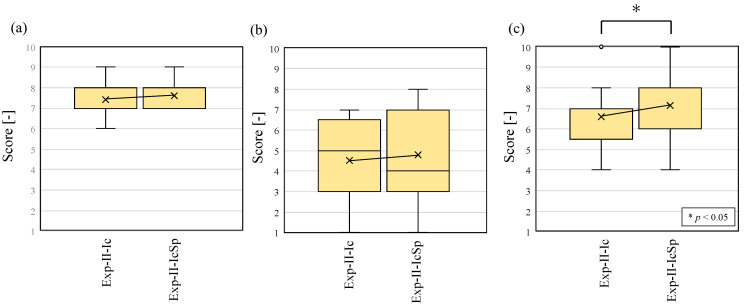
Results of subjective evaluation of questions 39 to 41 (* *p* < 0.05): (**a**) whether the subject was able to identify the location of the cohabitant (Q. 39), (**b**) whether the subject felt stressed from misleading information (Q. 40), and (**c**) whether the subject was able to comfortably carry out the pre-defined tasks (Q. 41).

The results of questions 39 to 41 are shown in Figure 17. First, the results of question 39 (Figure 17a) show that in both the Exp-II-Ic and Exp-II-IcSp experiments, the understanding of the location of the person in charge was high and did not differ from each other. The results of question 40 in Figure 17b show that both Exp-II-Ic and Exp-II-IcSp had a large variation, and although the stress felt when the environmental sound was wrong appeared to vary from individual to individual, there was no statistically significant difference between Exp-II-Ic and Exp-II-IcSp. Finally, with regard to the comfort felt by the subjects, the results of question 41 (Figure 17c) show a significant difference (*p* < 0.05) between Exp-II-Ic and Exp-II-IcSp, indicating that the improvement in recognition accuracy improved comfort.

The results of the last three questions showed no statistical differences between Exp-II-Ic and Exp-II-IcSp with regard to the identification of the cohabitant location and the stress felt by the subjects, but the final comfort level was found to have improved. This may be due to the fact that, in particular, the combination of both the ML-based icon and the spectrogram increased the information available to the test subjects on which to base their decision about the identity of the environmental sounds, and thus also gave them more degrees of freedom in their judgments. In this study, the advantages and disadvantages of both the ML-based icons and the spectrogram could be distinguished, and the improvement in comfort when they are used together was confirmed. The findings obtained in this study are expected to contribute to the development of environmental sound displays for DHH people in the future. As mentioned in the limitations of this paper (Section 3.3), the future development of hardware will contribute significantly to the improvement of technology to support people with hearing impairments.

## 6. Conclusions

With the aim of supporting DHH people in terms of environmental sound detection in their dwellings, evaluation experiments on ESR using a visualization device were conducted. The results showed that the proposed AR visual presentation system, which combines an ML-based icon that classifies the environmental sounds and a real-time dynamic display of spectrograms, improved the comfort of daily life in household spaces by combining not only the ML classification results but also the real-time display of spectrograms, some of which can be interpreted by non-experts after training.

## Figures and Tables

**Table 1:** Survey results of the current and previous interviews. The check marks are assigned to each of the references where the corresponding comments are described.

Category	Sub-Category	Coded Contents	Current Interview	Reference (ICCD 1995) [54]			Other References	
					Number of Respondent	Answered Contents		
General issue	Detecting evenets	Difficulty of detecting events	✔	―			✔	Jain et al., 2020 [42]
	Communication	Difficulty in communicating with others	✔	―			―	
	Reading text	Difficulty in reading text with a natural hearing impairment person	✔	―			✔	John et al., 2016 [57]
Interior issue	Auditory and vibration detection	Difficulty of waking up without alarm	✔	✔	45/180	Feel difficulty in waking up alone	✔	Matthews et al., 2006 [35] Bragg et al., 2016 [18]
				✔	66/180	Usually wake up by using vibration alarm	✔	Jain et al., 2020 [42]
	Auditory detection	Difficulty in detecting the state of cooking	―	✔	61/180	Feel difficulty in detecting boiling	✔	Jain et al., 2020 [42]
	Auditory detection	Difficulty of detecting status change of home appliances (such as kettles, microwave ovens, and smoke detectors)	―	―			✔	Matthews et al., 2006 [35] Mielke et al., 2015 [56] Bragg et al., 2016 [18]
	Auditory detection	Difficulty in detecting operation sound (such as vacuum cleaner or running water)	✔	✔	23/180	Feel difficulty in detecting running water	✔	Matthews et al., 2006 [35]
				✔	41/180	Feel difficulty in detecting to left washing machine finished	✔	Mielke et al., 2015 [56]
				✔	11/180	Feel difficulty in detecting to left vacuum cleaner on	―	
	Auditory detection	Difficulty in detecting rain	✔	―			―	
	Auditory detection	Difficulty of detecting knocking on doors	✔	―			✔	Matthews et al., 2006 [35] Jain et al., 2020 [42]
	Auditory detection	Difficulty of detecting transient sound like dropping things	✔	―			✔	Bragg et al., 2016 [18]
	Auditory detection	Difficulty of detecting someone coming to their home	―	✔	54/180	Feel difficulty in detecting someone come to home	✔	Mielke et al., 2015 [56] Bragg et al., 2016 [18] (detection of doorbell)
	Auditory detection	Difficulty of detecting someone’s replying voice	―	✔	32/180	Feel difficulty of detecting someone’s replying voice	✔	Jain et al., 2020 [42] (difficulty of detecting voices directed to me)
	Auditory detection	Difficulty of detectin baby crying	―	―			✔	Mielke et al., 2015 [56] Jain et al., 2020 [42]
	Visual detection	Alternative methods for event detection; use of visual information such as flashing lights	✔	―			✔	Jain et al., 2020 [42]
Exterior issue	Auditory detection	Difficulty in detecting information such as approaching vehicle sounds or someone’s call	✔	✔	72/180	Feel difficulty in detecting approaching vehicles and someone’s call	✔	Nakagawa et al., 2007 [55] Bragg et al., 2016 [18]
	Auditory detection	Difficulty in detecting platform or internal announcements or emergency broadcasts	―	✔	82/180	Feel difficulty in detecting platform or internal announcements or emergency broadcasts	✔	Bragg et al., 2016 [18]
	Auditory detection	Difficulty in detecting store clerk’s call of his and her name	―	✔	103/180	Feel difficulty in detecting hospital clerk’s call	―	
				✔	72/180	Feel difficulty in detecting clerk’s call of banks or post offices	―	
	Auditory detection	Difficulty in communicating with doctor in hospital	✔	✔	79/180	Feel difficulty in communicating with doctor in hospital	―	
	Auditory detection	Difficulty in communicating with deriver of taxi	―	✔	62/180	Drivers talk while facing forward, so you don’t realise they are talking	―	
				✔	31/180	Telling the driver where you are going is not understood	―	
	Auditory detection	Difficulty in hearing the sirens of police cars or ambulances	―	✔	21/180	Feel difficulty in hearing the sirens of police cars or ambulances when driving	✔	Mielke et al., 2015 [56] Bragg et al., 2016 [18] Jain et al., 2020 [42]
	Auditory detection	Difficulty in communicating with store clerk	―	✔	70/180	Feed difficulty in hearing the clerk’s explanation of products	―	
Interior and exterior issues	Auditory detection	Difficulty of detecting the sound of someone’s approaching footsteps	✔	―			✔	Jain et al., 2020 [42]
	Auditory detection	Difficulty in using telephone	―	✔	39/180	Feel difficulty in using telephone in case of calling ambulances	―	
	Auditory detection	Difficulty in obtaining information in case of disaster	―	✔	39/180	Feel difficulty in obtaining information in case of disaster	―	

**Table 2:** Twelve kinds of household environmental sounds examined in this study.

Categories	Sounds
Tonal sounds	Alarm Intercom Human voice (male) Human voice (female)
Transient sounds	Dropping purse Dropping plastic bottle Knocking on door Opening door Closing door Footsteps
Steady-state sounds	Running water on the sink Running water on the washbasin

**Table 3:** Overview of target rooms A, B, and C and the anechoic room.

	Structure of the Building	Area of Space	Measured Sounds
Room A	Wooden structure	33. 5 m^2^	Alarm Intercom Dropping plastic bottle Dropping purse Opening door Closing door Knocking on door Footsteps Running water from the sink Running water from the washbasin
Room B	Reinforce-concrete structure	9.1 m^2^	
Room C	Reinforce concrete structure	30.0 m^2^	
Anechoic room	Wooden panel structure	3.8 m^2^	Human voice (male) Human voice (female)

**Table 4:** Two different divisions of environmental sounds into 8 or 10 sub-categories.

Sounds	Categorization	
	Eight Types	Ten Types
Alarm	Alarm sounds	Alarm sounds
Intercom	Intercom sounds	Intercom sounds
Dropping purse	Dropping sound	Sound of a dropping purse
Dropping plastic bottle		Sound of dropping a plastic bottle
Opening door	Sound of opening/closing the door	Sound of opening the door
Closing door		Sound of closing the door
Knocking on door	Sound of knocking on the door	Sound of knocking on the door
Footsteps	Sound of footsteps	Sound of footsteps
Running water on the sink	Sound of runnning water	Sound of runnning water
Running water on the washbasin		
Human voice (male)	Human voice	Human voice
Human voice (female)		

**Table 5:** Structure of the confusion matrix.

		Predicted Class	
		Positive	Negative
True class	Positive	TP (True Positive)	FN (False Negative)
	Negative	FP (False positive)	TN (True Negative)

**Table 6:** Recognition accuracy of each condition of classification into 8 or 10 sub-categories.

	Environmental Sounds			Accuracy		Recall			Precision			F-Score	
8 Sub-Categories		10 Sub-Categories		8	10	8	10		8	10		8	10
A_8_	Alarm	A_10_	Alarm	0.70	0.59	0.78	0.78		0.88	0.88		0.82	0.82
B_8_	Intercom	B_10_	Intercom			0.75	0.75		0.75	0.75		0.75	0.75
C_8_	Dropping	C_10_	Dropping plastic bottle			0.86	0.6		0.67	0.75		0.75	0.67
		D_10_	Dropping purse			0.86	1		0.67	0.17		0.75	0.3
D_8_	Opening/Closing door	E_10_	Opening door			0.78	0.5		0.8	0.43		0.79	0.47
		F_10_	Closing door			0.78	0.29		0.8	0.55		0.79	0.38
E_8_	Knocking on door	G_10_	Knocking on door			0.67	0.67		0.91	0.91		0.77	0.77
F_8_	Footsteps	H_10_	Footsteps			0.67	0.67		0.36	0.53		0.47	0.59
G_8_	Running water	I_10_	Running water			0.5	0.5		1	1		0.67	0.67
H_8_	Human voice	J_10_	Human voice			1	1		0.73	0.73		0.84	0.84
I_8_	Silence	K_10_	Silence			0.81	0.81		0.58	0.58		0.68	0.68

**Table 7:** Recognition accuracy of each of the three tests.

Environmental Sounds	Accuracy			Recall				Precision				F-Score		
	Exp-1st	Exp-2nd	Exp-3rd	Exp-1st	Exp-2nd	Exp-3rd		Exp-1st	Exp-2nd	Exp-3rd		Exp-1st	Exp-2nd	Exp-3rd
Alarm	0.70	0.89	0.91	0.82	0.89	0.91		0.80	1.00	0.95		0.81	0.94	0.93
Intercom				0.82	1.00	0.98		0.93	0.98	0.96		0.87	0.99	0.97
Dropping				0.44	0.78	0.76		0.37	0.71	0.72		0.40	0.75	0.74
Opening/closing door				0.47	0.80	0.87		0.47	0.78	0.80		0.47	0.79	0.83
Knocking on door				0.53	0.80	0.80		0.62	0.90	0.92		0.57	0.85	0.86
Footsteps				0.80	0.93	0.98		0.82	0.91	1.00		0.81	0.92	0.99
Running water				0.91	0.98	0.98		0.93	0.90	0.94		0.92	0.94	0.96
Human voice				0.82	0.93	0.98		0.80	0.96	0.98		0.81	0.94	0.98

**Table 8:** Recognition accuracy of each of experiments Exp-II-Ic and Exp-II-IcSp.

Environmental Sounds	Accuracy		Recall			Precision			F-Score	
	Exp-II-Ic	Exp-II-IcSp	Exp-II-Ic	Exp-II-IcSp		Exp-II-Ic	Exp-II-IcSp		Exp-II-Ic	Exp-II-IcSp
Alarm	0.71	0.88	0.85	1.00		1.00	1.00		0.92	1.00
Intercom			0.74	1.00		0.90	1.00		0.81	1.00
Dropping			0.79	0.88		0.50	0.72		0.61	0.79
Opening/closing door			0.55	0.65		0.42	0.77		0.48	0.70
Knocking on door			0.45	0.82		0.88	0.96		0.59	0.89
Footsteps			0.29	0.50		0.78	0.50		0.42	0.50
Running water			0.84	0.90		0.84	0.95		0.84	0.93
Human voice			0.93	1.00		0.72	0.93		0.82	0.96

## Data Availability

The data that support the findings of this study are available from the corresponding author, T.A., upon reasonable request.
